# Predicament and outlook of China's math education

**DOI:** 10.1093/nsr/nwaa070

**Published:** 2020-04-17

**Authors:** Weijie Zhao

**Affiliations:** NSR news editor based, Beijing

## Abstract

**Abstract:**

Mathematics is the foundation of science and rational thinking. Math education for the younger generation is the fundamental project to upgrade the mathematical literacy and the creativity of the whole society. China's education system has long been different from that of Western countries. China has fostered many gold medal winners of the International Mathematics Olympiad, but is also criticized as lacking creativity. In this *NSR* forum on math education in China, educators of high schools and universities as well as researchers of different scientific fields gather to talk about the current predicaments and future developments of China's math education.

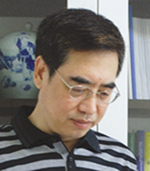

**Zenghu Li**

Mathematician; Professor of the School of Mathematical Sciences, Beijing Normal University, Beijing, China

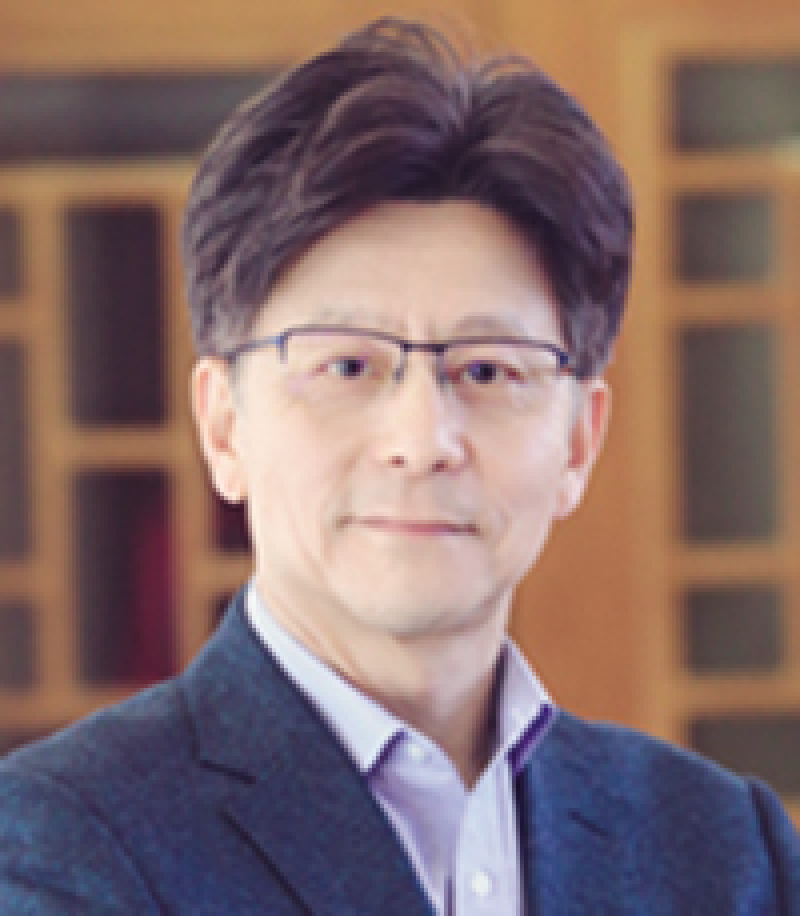

**Chao Tang**

Quantitative biologist; Director of the Center for Quantitative Biology, Peking University, Beijing, China

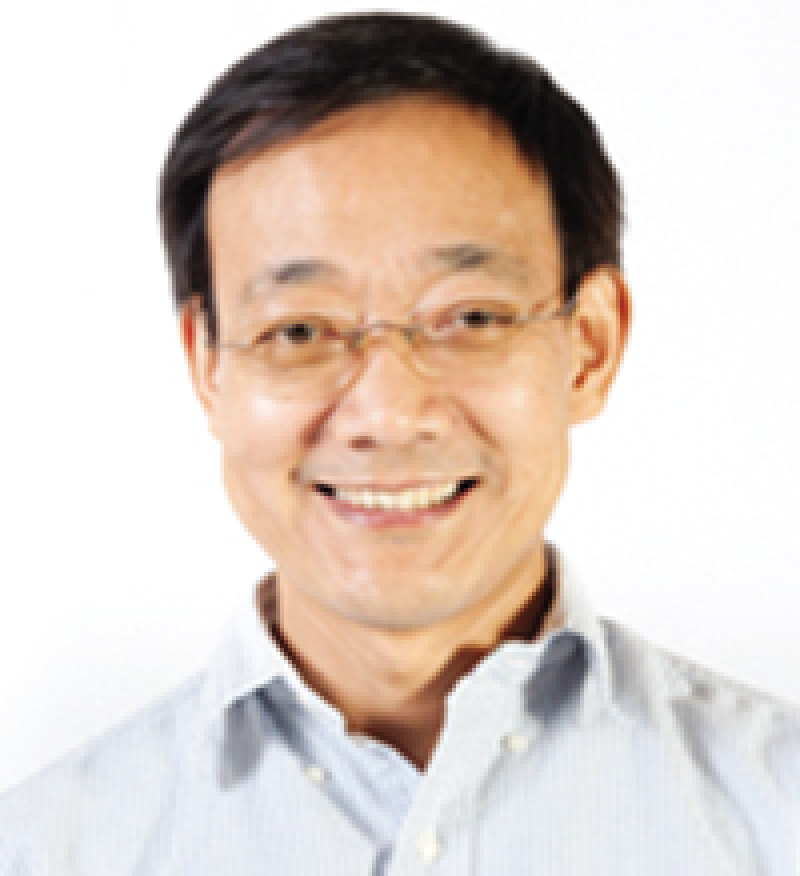

**Zhihong Xia**

Mathematician; Professor of Mathematics, Northwestern University, Evanston, USA and the Founding Chair of the Department of Mathematics, Southern University of Science and Technology, Shenzhen, China

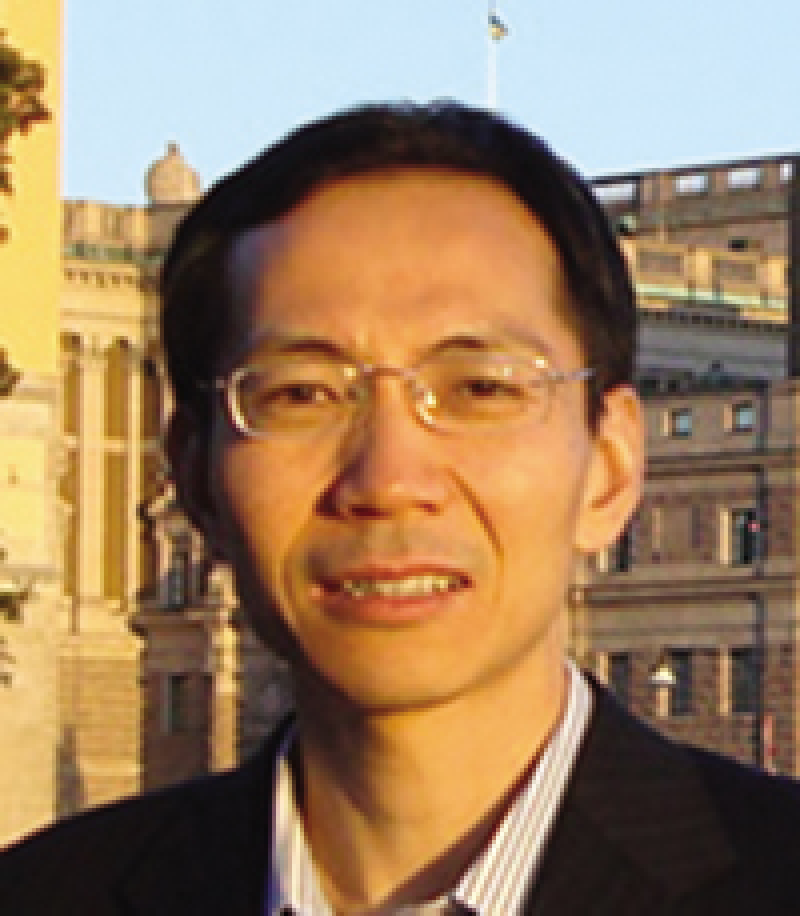

**Jinlong Yang**

Computational chemist; Professor of the School of Chemistry and Materials Science, University of Science and Technology of China, Hefei, China

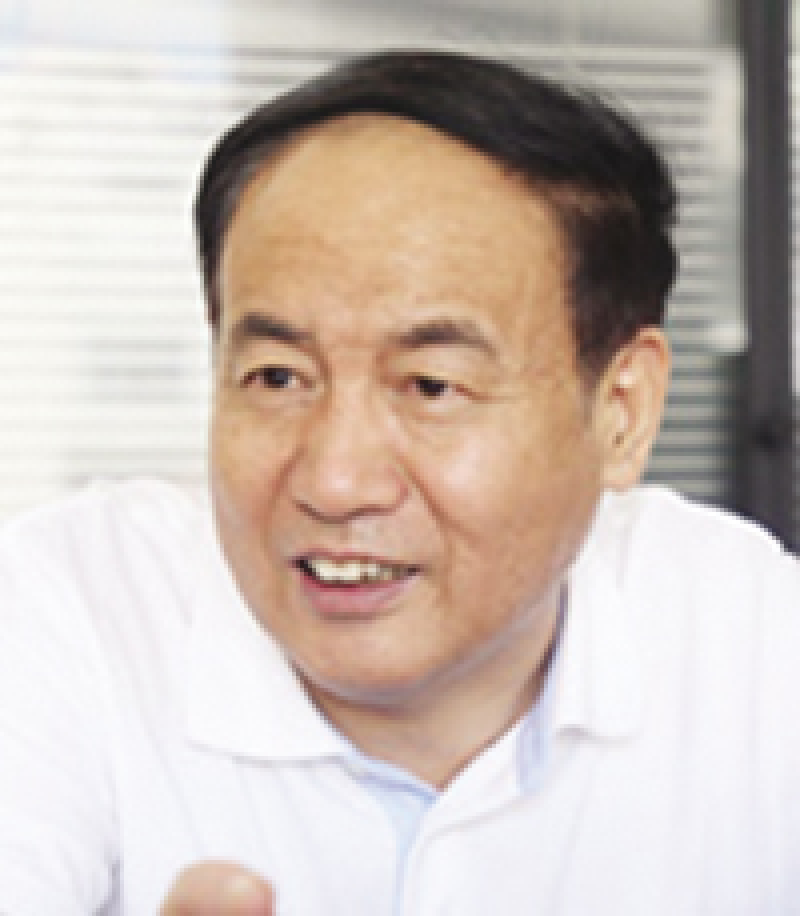

**Huawei Zhu**

Headmaster of Shenzhen Middle School, Shenzhen, China; Former leader and head coach of the national team of China for the International Mathematics Olympiad, China

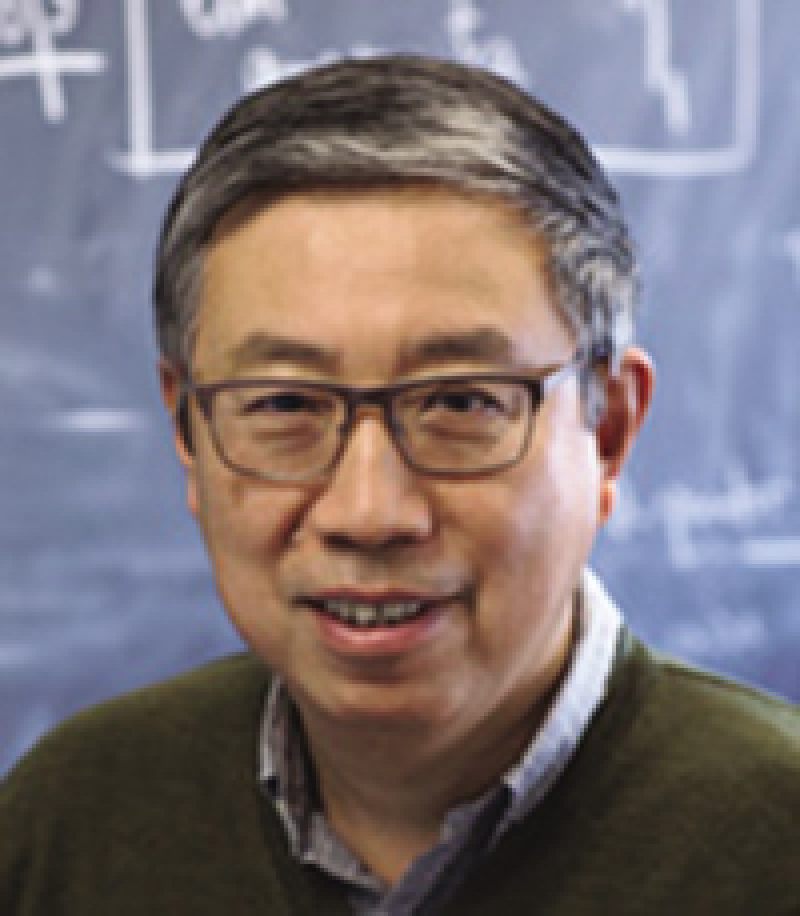

**Gang Tian (Chair)**

Mathematician; Professor of the School of Mathematical Sciences, Peking University, Beijing, China

## HIGH-SCHOOL MATH EDUCATION: OPTIONAL COURSES IN CHINA AND ADVANCED PLACEMENT COURSES IN THE USA

**Tian:** High school is a basic stage for math education. Let's start from here.

**Xia:** As I know, the US high-school courses are quite flexible. One can freely choose the courses you want to take, and one can choose the level of difficulty. If you do not want to learn any math courses in a certain semester at all, it is OK too. I guess the Chinese high-school courses are not as flexible as the American ones.

**Zhu:** In China, the courses of primary schools and middle schools are fixed, but there is some flexibility in high schools. There are three types of high-school courses: compulsory courses, distributional electives and optional courses. Before 2020, there were 16 optional math courses, including difference equations, number theory, cryptography, spherical geometry, symmetry and groups, etc. But this arrangement was not successful, because no high school had the ability to teach all the 16 topics. Starting from the autumn of 2020, many optional courses that were nearly impossible for high-school students to master will be canceled. But it also means that the flexibility will be decreased.

Besides the 16 optional courses, some high schools offer more optional courses. Shenzhen Middle School (SMS) offers advanced placement (AP) courses of calculus, statistics and linear algebra, as well as other optional math courses such as mathematical modeling and math contest. I know that, in the USA, there are more AP courses for high-school students.

**Li:** Among the three types of Chinese high-school courses, compulsory courses and distributional electives roughly cover the scope of the National College Entrance Examination (commonly known as Gaokao). But the optional courses are not tested in the Gaokao.

**Tang:** In the USA, AP courses are helpful for college applications and can be recognized as university credits. But the Chinese optional courses are not related to the Gaokao, so the schools, parents and students are not willing to spend time on them.

The optional courses are not tested in the Gaokao.

—Zenghu Li

**Zhu:** Exactly. In SMS, the optional courses are mostly taken by students who are going to universities abroad, but not the great majority who would take the Gaokao.

**Xia:** In the top US universities, some freshmen are able to take advanced math courses and some second-year students are able to take graduate courses, which is unimaginable for the Chinese students. I think these American students benefited a lot from the AP courses.

**Yang:** The basic physics and chemical courses in college require mathematical knowledge of calculus and linear algebra. In Chinese universities, most freshmen have no such knowledge so that we have to stop the specialized courses for one or two weeks, during which we supply them with the mathematical knowledge before we can come back to the main course. So I think the linkage of high school and university is not well organized in China.

This may greatly affect the students. If they encounter difficulties in the first specialized courses at university, they would lose confidence and interest in their major, and it would be very difficult for them to catch up in the following courses.

**Zhu:** In the 1980s, we tried to teach calculus in high schools. But there were critiques that high-school teachers were not well trained for calculus so that the students had to learn it again at university.

**Li:** There are different ways to improve high-school education so that graduates can accommodate their university studies quickly and smoothly. The AP courses of the USA and the prepas in France are typical examples. In France, most high-school graduates go to universities, while some of the best graduates go to the prepas, where they learn calculus and other basic courses for 2 years before entering the Grande Ecoles. This mode is very successful and has fostered much talent for France.

I think, for China, the AP mode might fit our situation better. Actually, we have been attempting to set up AP courses for several years. There is still a long way to get them properly evaluated in Gaokao and make the credits recognized by the universities. But it is worthwhile to push forward with the project.

## EXAMINATION-ORIENTED EDUCATION: THE WASTED AGE 17

**Tang:** Some students in Peking University told me that they did not learn anything new in the last year of high school. They feel that that year was wasted.

**Zhu:** China's high-school education is Gaokao-oriented, which has raised some problems.

First, we have to teach and learn what is tested in the Gaokao, no more and no less. After decades of practice, the styles of Gaokao questions have been totally fixed. Every year, the students face questions of the same number, the same types and in the same order. To get high marks in the Gaokao, all high schools throughout China teach almost exactly as the Gaokao requests and the students are trained month after month to solve the same types of questions as quickly and accurately as possible.

Second, this training kills students’ interests in math. Students accept large amounts of repeated training to form ‘conditional reflexes’ to the questions. But, through this training process, students who were initially interested in math cannot enjoy the joy of acquiring new knowledge and independent thinking, and that will definitely ruin their interest.

Third, students selected by exam-oriented training usually cannot get very far. There is a middle school in Shenzhen that is very good at training for the High School Entrance Examination. Every year, many students of this middle school enter the high school of SMS, but most of them cannot perform well in the high school. I think it is the same for the Gaokao. Many Gaokao top scorers become ordinary at universities. Meanwhile, many students selected by math contests keep being the best.

Fourth, and most importantly, the last year of high school is wasted. The students are 17 years old in their last year of high school. This is the best age to absorb and comprehend new information—the best age to think about life and to build their own philosophies. But all Chinese students spend their entire 17-year-old time in repetitive training for the Gaokao, which greatly harms their creativity as well as the future of the whole nation.

All Chinese students spend their entire 17-year-old time in repetitive training for the Gaokao, which greatly harms their creativity as well as the future of the whole nation.

—Huawei Zhu

**Tang:** Very good point. We cannot change China's education without reforming the Gaokao.

**Zhu:** I have a simple suggestion for the Gaokao: cancel the 12 choice questions in the math examination. Currently, there are 22 questions in the Gaokao math examination, including 12 choice questions, 4 fill-in-the-blanks and 6 comprehensive questions. The 12 choice questions, whose types and orders are completely fixed, are actually unnecessary. We should first cancel the choice questions, then cancel the fill-in-the-blanks questions and leave only the comprehensive questions. This could endow the students with more space for independent thinking and stop selecting students by repetitive training.

Actually, many foreign math examinations, as well as the Gaokao math examination in the late 1970s, do not contain choice questions and these exams are effective enough to select talent.

But, the reality is, it is nearly impossible to change the Gaokao. Changing the style of a single question may lead to great repercussions from the parents, not to say the cancelation of all the choice questions.

**Xia:** Chinese students are really good at exams. Take the GRE advanced math test as an example. Among applicants to Northwestern University, Chinese students mostly rank at >90% in the exam. But, once the ETS, which administers the exams, changed the styles and inventories of the questions, their test scores mostly fell to 70% and above. Several years after the changes, their test scores came back again to 90% and above. So we can tell that the scores of Chinese students cannot truly reflect their preparedness for math, but rather their preparedness for taking exams. Maybe we should change the styles and types of questions in the Gaokao every year, so that repetitive training would not work.

## BEYOND THE GAOKAO: SELECT STUDENTS WITH SPECIAL TALENTS

**Tian:** I agree that repetitive training for the Gaokao cannot foster students’ interest of math. In the School of Mathematical Sciences of Peking University, the best students are often selected by math contests or the Independent Recruitment program. These students often have solid basic skills and a real interest in mathematics. Meanwhile, the scores in the Gaokao cannot predict the future development of students.

Currently, the government has made a new policy for entrance to university. But I think these special talent-selecting programs are necessary. We have to make a balance between fairness and talent selection.

**Tang:** The students entering universities through the contests are strong in a certain subject, but they are often weak in other subjects.

**Zhu:** Actually, in recent years, for most contest students, it is impossible to be too weak in the other subjects because they have to pass the general examinations and the high schools dare not bet their future on a single contest.

On the other hand, if someone is a real genius in mathematics, we should allow him or her to fully develop his or her talent. We should not waste his or her time in repetitive training in other subjects. The SMS cooperated with Huawei to set the SMS-Huawei Award for Special Talents. We give special education and offer very flexible environments to the special talents. If we can better support these students, they may become the future masters in diverse fields. We should not ruin their talents by forcing them to learn every subject.

**Xia:** Among all the Chinese students, only a small number are allowed to develop their interests by contests. Maybe we should change the system and allow all students to enjoy their interests to some degree, instead of training all students by a single standard.

There is more flexibility in the USA. Students can select their own courses starting from middle school. When they have graduated from high school, the best students have taken many AP courses and their math skills can be 2 years ahead of their grademates.

**Tang:** Another advantage of this kind of flexible system is that the students who are not good at mathematics would not lose confidence and be afraid or unable to get into universities, because they can develop other skills for university application. This is helpful for the development of the students’ confidence and self-recognition.

When I was a professor in the USA, I regularly received e-mails from high-school students volunteering to work in my lab in the summer. They wanted to experience biological research and decide whether to go into this field in the future. I have been back in China for many years, but have not received any such e-mails from Chinese high-school students.

**Xia:** I received such e-mails too. But I think lab experiences are helpful for college application in the USA and that is one of the reasons why American students are doing so.

**Tian:** That's right. If it is helpful for the Gaokao, Chinese students would like to go to the labs too. Actually, the Yingcai Program organized by China Association for Science and Technology (CAST) is encouraging high-school students to experience scientific research in universities. But the scale of this program is not big, with only about 120 students participating in this year's math division. Students are not very attracted because it is not directly related to the Gaokao.

We have to make a balance between fairness and talent selection.

—Gang Tian

**Yang:** I am also involved in the chemistry division of the Yingcai program. This program is currently organized in 20 universities and only available for high-school students in the cities where these 20 universities are located. Once enlisted in the program, they need to go to the universities once every two weeks.

Meanwhile, there have been more students applying for this program in the last two years, partly as a result of more students being willing to go to universities abroad, and this experience may be helpful.

**Xia:** Is it possible to extend this program to more cities and more universities? Maybe we can allow the universities to decide the details by themselves.

**Yang:** It is not easy at this stage. This program is funded by CAST and not all universities are able to participate unless they have the professors qualified and willing to do such work.

## THE IMPORTANCE OF MATHEMATICS FOR LOGIC THINKING AND MULTIPLE SCIENCE DISCIPLINES

**Tian:** Advanced mathematical knowledge is not needed in the daily lives of ordinary people. Then what is the importance of mathematics? What are the basic mathematical skills everyone should master?

**Tang:** I think university students, no matter what their major is, should learn calculus, linear algebra and statistics. But, of course, if they have majored in literature or art, the courses can be relatively easy, introducing just the concepts.

**Xia:** I think math training is not about calculus, trigonometric functions or any specific subjects themselves. It is about the way of thinking. It is the training of reasoning and logic deductions, telling you how you can rationally go from A to B, B to C, but cannot add a D from nowhere. It is also the training of abstraction, enabling us to imagine beyond the 3D world. Many people do not need calculus in their lives, but they do need to think logically and that's what math education can provide.

**Li:** Mathematics is everywhere, even in our daily lives. As an example, the principle of ‘randomized double-blind and large samples’ in drug tests has been talked about quite a lot recently. The principle is justified by the theory-of-independence tests, which is already included in our high-school mathematics as a part of the humanistic quality-oriented education.

**Yang:** Mathematics is becoming more and more important in many fields. For instance, chemistry was once an experimental science but, as its theory keeps developing, we are beginning to understand chemical reactions at the level of quantum mechanics so that many physical and mathematical tools are needed, such as the Schrodinger equation and the related mathematical tools.

We are also beginning to use artificial intelligence in reaction analysis and design, and the related algorithms are also math problems.

**Tang:** It is the same for biology. Traditionally, biology is descriptive. But, as the amount of data gathered and phenomena observed fast increasing, we cannot figure out the underlying patterns and principles without the help of mathematical tools. Now, informatics, statistics and artificial intelligence are all used in biological research.

More importantly, the life system is so unique that maybe we have not found the mathematical language that is appropriate to describe it. Newton created calculus when researching planetary motion. Maybe we should cooperate with mathematicians to create the math language for biology so that we can fundamentally promote the level of biological research.

Many people do not need calculus in their lives, but they do need to think logically and that's what math education can provide.

—Zhihong Xia

**Tian:** That is an important point. A mature science should have its own basic theories, on which we can make predictions. Will there be such theories in biology?

**Tang:** I believe so. It is now a golden time to use mathematical tools in biology. There has been work to understand the robustness, function and design principles of biology systems using math tools and I believe there will be more and more work like this.

**Xia:** The algorithms of big data and artificial intelligence are actually purely mathematical problems. For many scientific disciplines, such as computer science and chemistry, when they were first founded, they did not seem to rely much on mathematics but, as these fields developed, we suddenly realized that mathematics is so important for them; it can play a fundamental role. I think that is why mathematics and mathematics education are so crucial.

Life system is so unique that maybe we have not found the mathematical language that is appropriate to describe it.

—Chao Tang

## MATH EDUCATION IN UNIVERSITIES

**Tian:** Can your graduate students meet the math requirement of your discipline? Or is China's university education capable of cultivating qualified future scientists?

**Tang:** My graduate students come from the schools of life sciences, mathematics and physics. The students who majored in mathematics or physics are often well equipped with mathematical skills, but the students who majored in life sciences usually lack math skills. But, of course, some students who majored in life sciences took additional math courses out of personal interest in college so that they are also good at math.

**Yang:** I have a background of physics and now research on computational chemistry. I have graduate students who majored in physics or chemistry in college. Among them, the students who majored in physics have better math skills than the students who majored in chemistry.

However, I have to mention that students who graduated from the School of Chemistry in our university, which is the University of Science and Technology of China (USTC), often have adequate math skills because USTC has been emphasizing the significance of mathematics and physics since its foundation. In USTC, the math courses of the School of Chemistry are almost the same as those of the School of Physics. Students have to take multiple math courses for 2.5 years. Some students complain that they have mathematics and physics courses that are too heavy, but we always tell them that it is beneficial for their future.

Moreover, we have made some adjustments to the chemistry courses in USTC. Students should take theoretical chemistry and quantum chemistry in the second year, thus they would be familiar with the modern chemistry concepts and be aware of the importance of mathematics at the early stage. That would encourage them to pay more attention to mathematics.

But most other Chinese universities have not realized this problem. Except for students who majored in physical chemistry, few students would take quantum chemistry and related math courses at the undergraduate stage.

**Tang:** This is insightful. Students in biology often lack the motivation to learn mathematics because, in their minds, mathematics has no relevance to biology. If we can give more biology-related examples in our mathematics courses, students may better realize the importance of mathematics.

At Peking University, we established a special class of Integrated Science, which tries to integrate physics and mathematics with life sciences. We have researchers who are using physical or mathematical tools to study life sciences to give courses to this special class. The goal is to foster the next generation of quantitative biologists.

**Li:** To emphasize mathematics in teaching, we should first emphasize mathematics in research. If the professors are cooperating with mathematicians or doing research using mathematical tools, they will naturally convey the significance of mathematics to their students. That is to say, the quality of the lecturers is important.

Students should take theoretical chemistry and quantum chemistry in the second year, thus they would be familiar with the modern chemistry concepts and be aware of the importance of mathematics at the early stage.

—Jinlong Yang

**Tian:** The next question is how can the quality of math education in universities be improved?

The quality of lecturers is actually important. In the early 1980s, China's mathematical research had a considerable gap compared with the foreign countries, but there were a group of college teachers who had received strict training on the basic skills of mathematics and they were able to foster a number of outstanding students who majored in mathematics.

**Xia:** Discussing classes dominated by students is a good way to foster students’ interest in mathematics. As I know, there are such courses in both Peking University and the Southern University of Science and Technology. Several students decide what to discuss and the teacher would join and guide the discussion.

**Yang:** USTC also has a similar discussion class, which is a one-year course named Science and Society. In each class, one teacher guides the discussion of around ten students. In the discussion classes with a topic of mathematics, students can fully experience the significance of mathematics in society and diverse fields.

**Xia:** Universities love to invite famous scientists to give lectures, but many of these famous scientists might not be good speakers, particularly to an undergraduate audience. Often, students cannot benefit much from these talks. So, when we decide whom to invite, maybe we should consider not only the lecturer's scientific achievement and prestige, but also his or her ability to give talks, to inspire our students.

## MATHEMATICAL RESEARCH IN CHINA

**Li:** Mathematical research in China has been developing rapidly in the past two decades. A number of overseas Chinese mathematicians have returned to China to teach and have made very important contributions to the development of mathematics in China.

**Tian:** That's right. Our research level has been advanced greatly, and we are beginning to foster the next generation of mathematicians in China. Chinese mathematicians made progress in the fields of geometric analysis, algebraic geometry, complex geometry and number theory. It is hopeful to establish some ‘Chinese Schools’. But we should notice that innovative world-leading work is still lacking.

**Tang:** Lack of innovativeness is a common problem for many research fields in China. What is the root of this phenomenon? Is it because of our education system, research system or talent selection and retention system?

**Xia:** All these factors are relevant. But I think, as we have talked about, the exam-oriented education system is not good at selecting and cultivating innovative talent. Yes, the system is good at cultivating skilled workers conforming to a uniform standard, but not innovative talents with independent thinking.

On the other hand, I think the Chinese government should not require all math professors in all universities to do research. Many professors are not qualified and do not want to do research. We should allow them to focus their efforts on education. Low-quality disingenuous ‘research’ may do more harm to the overall environment of China's mathematical research.

**Tian:** Currently, several Chinese universities, including Peking University and USTC, are able to cultivate high-quality undergraduate students who have majored in mathematics. But our universities are not attractive enough compared to the universities of Western countries. Our best undergraduate students would like to go abroad for further education and there are few foreign students willing to study in China, thus influencing the overall quality and cultural diversity of China's graduate students and postdoctoral researchers.

**Xia:** Many Chinese students go to the USA for PhD degrees in mathematics. But some of them are not truly interested in mathematics and would be actively seeking to transfer to other fields such as finance, business and industrial engineering. For American students, if they apply for PhD programs in mathematics, then they are truly interested in mathematics and want to pursue careers in mathematics.

## CONCLUSION AND SUGGESTIONS

**Tian:** As a conclusion of the discussion, let's give some suggestions to China's math education. Headchair Zhu has suggested canceling the 12 choice questions in the Gaokao. Besides that, I think maybe we should give more autonomy to the universities.

**Xia:** That's right. We should allow the universities to run in their own styles. That would bring more diversity and creativity.

**All:** Yes. We all agree on the two suggestions: to reform the Gaokao starting from eliminating the choice questions, and to give the universities more autonomy.

